# Caution for psychiatrists: malignant hyperthermia risks with the anesthetic agent succinylcholine (Suxamethonium) during electroconvulsive therapy

**DOI:** 10.1186/s12888-024-05846-5

**Published:** 2024-06-04

**Authors:** Masaki Nakano, Michitaka Funayama, Taketo Takata, Riko Wakisaka, Genki Koyama, Akihiro Koreki, Takuto Ishida, Hiroyuki Uchida, Masaru Mimura

**Affiliations:** 1 Department of Neuropsychiatry, Ashikaga Red Cross Hospital, 284-1 Yobe, Ashikaga-city, Tochigi 326-0843 Japan; 2 Department of Neuropsychiatry, Keio University School of Medicine, Shinjuku, Tokyo 160-8582 Japan; 3 Department of Psychiatry, National Hospital Organization Shimofusa Psychiatric Medical Center, Chiba, 266-0007 Japan; 4 Tokyo Metropolitan Matsuzawa Hospital, Tokyo, 156-0057 Japan

**Keywords:** Malignant hyperthermia, Electroconvulsive therapy, Succinylcholine, Suxamethonium, Depression, Serotonin syndrome, Neuroleptic malignant syndrome

## Abstract

**Background:**

Malignant hyperthermia is a potentially lethal condition triggered by specific anesthetic drugs, especially a depolarizing muscle relaxant of succinylcholine (Suxamethonium). Despite the frequent use of succinylcholine with electroconvulsive therapy (ECT), there has been no reported case of potentially lethal malignant hyperthermia following ECT. In addition, the time interval between the administration of succinylcholine and the onset of malignant hyperthermia has not been outlined in the context of ECT.

**Case presentation:**

We present the case of a 79-year-old woman suffering from severe depression, who experienced severe malignant hyperthermia due to succinylcholine administration during an ECT session. She presented with a high fever of 40.2 °C, tachycardia of 140/min, hypertension with a blood pressure exceeding 200 mmHg, significant muscle rigidity, and impaired consciousness. These symptoms emerged two hours after ECT, which occurred in a psychiatric ward rather than an operating room, and reached their peak in less than 24 h. She was given 60 mg of dantrolene, which quickly reduced the muscular rigidity. Subsequently, she received two additional doses of 20 mg and 60 mg of dantrolene, which brought her fever down to 36.2 °C and completely eased her muscle rigidity within two days after ECT.

**Conclusions:**

This is the first reported case of potentially lethal malignant hyperthermia after ECT. In addition, it highlights the delayed onset of malignant hyperthermia following an ECT procedure, emphasizing the necessity for psychiatrists to recognize its onset even after the treatment. In the light of potentially lethal consequences of malignant hyperthermia, it is critically important for psychiatrists to closely monitor both intraoperative and postoperative patient’s vital signs and characteristic physical presentations, promptly identify any symptomatic emergence, and treat it immediately with dantrolene.

## Background

Malignant hyperthermia is a sudden and serious medical condition triggered by the use of volatile anesthetic inhalants or depolarizing muscle relaxants, such as succinylcholine (Suxamethonium), commonly employed during electroconvulsive therapy (ECT). Without prompt and effective intervention, the progression of malignant hyperthermia can lead to fatal outcomes, with a mortality rate of 9.5% [1, 2]. Dantrolene is recognized as the primary drug specifically designed to treat malignant hyperthermia [1] and should be administered quickly when this disorder is suspected. Considering succinylcholine’s regular use in ECT, there is a potential risk of malignant hyperthermia on ECT. Despite the widespread use of succinylcholine in ECT, only one very mild case of malignant hyperthermia occurring during or after ECT has been reported [3]. There is recognition among psychiatrists that succinylcholine may induce malignant hyperthermia. However, given that there is only one documented instance of its actual onset and considering that malignant hyperthermia is more commonly recognized among anesthesiologists than among psychiatrists, it is likely that some psychiatrists may not routinely consider this potential side effect. Additionally, the precise time interval between the ECT and the onset of malignant hyperthermia in the previous case was unclear [3], as was the question of whether the psychiatrist was the first to identify its onset.

We report a case of potentially lethal malignant hyperthermia that manifested in our psychiatric ward rather than in the operating room, occurring two hours after ECT which was performed with the use of succinylcholine. In this case, the patient exhibited abnormal vital signs, such as severe hyperthermia, as well as acute muscle rigidity and compromised consciousness. This case illustrates the delayed onset of malignant hyperthermia following an ECT procedure, emphasizing the need for psychiatrists to be vigilant in identifying its emergence within psychiatric wards.

## Case presentation

### Medical history

The patient is a 79-year-old female who experienced persistent insomnia starting at the age of 50, which lasted for five years. At the age of 55, she received a diagnosis of major depressive disorder at a psychiatric clinic. She was prescribed tricyclic antidepressants, which significantly alleviated her symptoms in six months.

At 76, she experienced a significant emotional disturbance following the sudden death of a close friend. This incident, which occurred 24 days before her first hospital admission, triggered severe anxiety symptoms: heart palpitations, restlessness, and an overwhelming fear of meeting a similar fate, leading her to wander aimlessly. She also exhibited agitated behavior, continuously pacing around her room. Additionally, she experienced sleep disturbances, a significant decrease in appetite, and cognitive decline, resulting in difficulty comprehending the contents of television programs. These symptoms prompted her to seek medical attention at our facility precisely two weeks before her subsequent hospitalization. According to DSM-5 diagnostic criteria standards, she was diagnosed with major depressive disorder, with a Montgomery-Åsberg Depression Rating Scale (MADRS) score of 53, indicating severe depression [4]. Outpatient treatment initiated with duloxetine (20 mg), lorazepam (1 mg), and lemborexant (5 mg). However, due to a lack of improvement and increased suicidal thoughts, she was admitted to our psychiatric unit.

### Malignant hyperthermia

Upon hospitalization, her medication was discontinued except for lemborexant (5 mg) orally. On the second day of hospitalization, ECT was administered due to the patient’s unresponsiveness to medication and intense suicidal ideation coupled with agitation. Notably, the patient’s physical condition, including vital signs and a Body Mass Index of 18.6 kg/m², had been entirely normal before the anesthesia induction. Anesthesia induction was carried out using 150 mg of sodium thiopental, an ultra-short-acting barbiturate, and 50 mg of succinylcholine, an ultra-short-acting depolarizing muscle relaxant. Additionally, 0.5 mg of atropine, a muscarinic antagonist, 20 mg of landiolol hydrochloride, an ultra-short-acting β-blocker, and 1 mg of nicardipine, a calcium channel blocker, were administered to manage side effects associated with ECT, such as excessive salivation, tachycardia or bradycardia, and elevated blood pressure. ECT procedures were executed without any issues. Her postoperative consciousness improved without complications, reaching a Glasgow Coma Scale score of 13 (Eye, 3; Verbal, 4; Motor, 6).

However, two hours post-ECT, her Glasgow Coma Scale deteriorated to 7 (Eye, 1; Verbal, 1; Motor, 5). At the same time, she exhibited marked muscular rigidity in her limbs, along with mydriasis in both eyes and diminished light reflexes. Given the potential for a brain disorder, a computed tomography scan and magnetic resonance imaging of brain were performed, which revealed no abnormalities. Blood tests showed elevated white blood cell counts to 24,400/µL, but no other abnormal findings. After analyzing the electroencephalogram obtained the same day, we found that the amplitude was markedly reduced without any detectable epileptic waveforms, which indicated an impaired state of consciousness. Approximately seven hours post-ECT, her vital signs became abnormal: her temperature rose to 39 °C, heart rate increased to 140 beats per minute, and systolic blood pressure sharply surged above 200 mmHg. To counter these abnormalities, strategies including continuous intravenous hydration, temperature management, and acetaminophen infusion were employed. Simultaneously, nicardipine, a calcium channel blocker, was used to treat hypertension. However, on the subsequent day post-ECT (the third day of hospitalization), 21 h after the ECT, her temperature further increased to 40.2 °C, and her Glasgow Coma Scale deteriorated to 6 (Eye, 1; Verbal, 1; Motor, 4), with ongoing muscular rigidity. Symptoms of dehydration and hyponatremia, resulting from sustained hyperthermia, were noted, as evidenced by elevated serum creatinine at 1.42 mg/dL, urea nitrogen at 33.9 mg/dL, and a reduced sodium level of 114 mEq/L. Furthermore, due to decreased consciousness resulting from hyponatremia, aspiration pneumonia developed as a complication. These conditions were managed with enhanced fluid resuscitation and the commencement of broad-spectrum antibiotics, specifically piperacillin-tazobactam at 3.375 g every six hours. A concurrent metabolic acidosis due to dehydration-induced circulatory inadequacy and a counteracting respiratory alkalosis were inferred from arterial blood gas results, which showed pH 7.490, PO2 65.9 mmHg, PCO2 24.2 mmHg, Base Excess − 3.4, HCO3- 18.2 mmol/L, and Lactate 3.2 mmol/L.

These clinical signs, including a rapid rise in body temperature, extensive muscular rigidity, and inconsistent sinus tachycardia, corresponded with the diagnostic criteria for malignant hyperthermia as defined by Larach et al. 1994 [5]. It is noteworthy, however, that the peak creatine kinase level, a key marker for malignant hyperthermia, displayed only a slight increase at 171 U/L on the third day of hospitalization (with the normal upper limit being 80 U/L) [6].

### Dantrolene administration

To treat malignant hyperthermia, dantrolene (60 mg) was intravenously introduced 23 h following the ECT on the third hospital day. Remarkably, following the dantrolene administration, muscular rigidity notably decreased within an hour, and her temperature dropped to 38 °C within four hours. Additional doses of 20 mg and 60 mg of dantrolene were administered on the third and fourth hospital days, respectively, led to a decrease of her temperature to 36.2 °C and the complete resolution of muscular rigidity. Subsequently, dehydration, its associated hyponatremia, and aspiration pneumonia were all successfully resolved. The state of her consciousness showed significant improvement, reaching a Glasgow Coma Scale score of 11 (Eye: 3; Verbal: 4; Motor: 4) on the seventh hospital day. This improvement coincided with an enhanced electroencephalogram displaying alpha waves, denoting lucid consciousness. No side effects associated with dantrolene were detected. By the seventh hospital day, she demonstrated the capability for basic conversations and began feeding on the seventeenth hospital day. Her depressive symptoms improved without the need for additional ECT or psychotropic interventions, and no residual cognitive deficits were observed. She was discharged from our institution on the thirty-second hospital day.

## Discussion and conclusions

This case represents the first instance of severe malignant hyperthermia after ECT. Compared with the previous report of malignant hyperthermia after ECT [3], our case is deemed more severe as the fever persisted for several days and was accompanied by impaired consciousness, which required the administration of dantrolene. Our case also highlights the delayed onset of malignant hyperthermia following an ECT session, emphasizing the need for psychiatrists to be vigilant in identifying its emergence in psychiatric wards. The symptom relief provided by dantrolene underscores its pivotal role in the therapeutic approach to malignant hyperthermia.

### Difference in symptoms between malignant hyperthermia, serotonin syndrome, and neuroleptic malignant syndrome

In this case, the patient exhibited fever, altered consciousness, and limb muscle rigidity, symptoms also observed in serotonin syndrome [7] as well as neuroleptic malignant syndrome [8]. Considering the differential diagnosis of serotonin syndrome is crucial, especially since the patient had been undergoing duloxetine treatment until two days before the ECT session. Given its half-life of 10–12 h (Knadler et al., 2011) [9], duloxetine’s effectiveness at the receptor level would have been present, albeit quite low, during the ECT procedure. Serotonin syndrome is a serious complication that can occur as a result of treatment with selective serotonin reuptake inhibitors, serotonin-norepinephrine reuptake inhibitors, tricyclic antidepressants, monoamine oxidase inhibitors, and other serotonergic medications (Mason et al. 2000) [10]. The syndrome is characterized by the sudden onset of cognitive/behavioral changes (confusion, agitation, lethargy, and coma), autonomic instability (hyperthermia, tachycardia, diaphoresis, nausea, vomiting, diarrhea, and dilated pupils), and neuromuscular changes (myoclonus, hyperreflexia, rigidity, and trismus) [10]. The present case exhibited coma, autonomic instability, and rigidity following the administration of a serotonin-norepinephrine reuptake inhibitor until two days before, potentially meeting the criteria for serotonin syndrome diagnosis [11]. Despite this, the likelihood of serotonin syndrome diagnosis in this case is quite low, primarily due to the timing of the patient’s symptoms and the notable efficacy of dantrolene. Firstly, it is generally unlikely for serotonin syndrome to emerge post-discontinuation of antidepressants [11]. Mason et al. (2000) reported that 74.3% of serotonin syndrome cases typically develop within 24 h of initiating medication, overdosing, or changing dosage [10], due to excessive serotonergic activation in both central and peripheral nervous system receptors [11]. Conversely, only 12.8% of cases emerge after 48 h [11]. Additionally, many cases of the serotonin syndrome typically resolve within 24 h after discontinuation of serotonergic drugs [11]. Secondly, the significant effectiveness of dantrolene does not indicate serotonin syndrome; instead, it strongly suggests a diagnosis of malignant hyperthermia. Dantrolene is recognized as the primary drug specifically designed to treat malignant hyperthermia [1], while Jones et al. (2005) highlight its unproven effectiveness in treating serotonin syndrome [12]. Additionally, this case lacked some symptoms that are frequently observed in serotonin syndrome, such as diarrhea, vomiting, and myoclonus [10, 11, 13]. The significant effectiveness of dantrolene treatment in this case may also indicate the improbability of neuroleptic malignant syndrome, as White et al. (2000) noted the limited therapeutic efficacy of dantrolene in neuroleptic malignant syndrome [14]. Conversely, malignant hyperthermia can readily explain this case and is the most plausible explanation, particularly considering the onset, which occurred immediately after the administration of the anesthetic agent succinylcholine during the ECT session, and the pronounced response to dantrolene. Mydriasis, which is one of the symptoms of serotonin syndrome [11, 13], can also be present in cases of malignant hyperthermia (Larach et al., 2006; Sheila et al., 2014; Sold et al., 1986) [15–17]. Thus, we diagnosed this patient with malignant hyperthermia rather than serotonin syndrome.

Regarding the distinction between malignant hyperthermia and neuroleptic malignant syndrome, historically, the similar symptomatology of these conditions has led to theories of a shared pathogenetic mechanism [18]; however, this hypothesis has not been definitively confirmed. Neuroleptic malignant syndrome and serotonin syndrome are believed to originate neurogenically rather than myogenically [11, 19], while the pathogenesis of malignant hyperthermia has been shown to be myogenic, specifically involving an abnormal release of Calcium from the sarcoplasmic reticulum [1]. Symptomatically, serotonin syndrome presents distinctive symptoms not typically found in malignant hyperthermia, including altered consciousness, behavioral changes, myoclonus, hyperreflexia, diarrhea, and vomiting [10, 11, 13, 20]. Rosenberg et al. (2015) observed no cross-susceptibility between malignant hyperthermia and neuroleptic malignant syndrome [1], and a review by Ortiz et al. (2020) found no genetic association between malignant hyperthermia, serotonin syndrome, and neuroleptic malignant syndrome [18].

However, in terms of treatment methods, there is some overlap between malignant hyperthermia, serotonin syndrome, and neuroleptic malignant syndrome. Immediate discontinuation of responsible agents and hydration are common practices in all of these conditions. Dantrolene administration is often performed as well. Psychiatrists may have mistakenly diagnosed malignant hyperthermia during ECT treatment as serotonin syndrome or neuroleptic malignant syndrome and administered dantrolene for treatment. Ironically, this misdiagnosis could have led to clinical improvement despite the error. The scarcity of reported cases of malignant hyperthermia during ECT treatment has been acknowledged in prior research [21, 22]. Some researchers have speculated that some ECT-related fatalities might have resulted from undetected malignant hyperthermia [22]. Alternatively, in our opinion, another explanation could be the misdiagnosis of malignant hyperthermia as serotonin syndrome or neuroleptic malignant syndrome by psychiatrists.

Still, it remains crucial to always consider the possibility of malignant hyperthermia during and after ECT, especially considering that psychiatrists more commonly encounter conditions like neuroleptic malignant syndrome and serotonin syndrome, which often take precedence in differential diagnoses. A misdiagnosis could lead to a delay in appropriate treatment due to differences in the discontinuation of triggering agents and the effectiveness of dantrolene.

### Characteristics of malignant hyperthermia in this potentially lethal case

Our case did not manifest a high level of creatine kinase, masseter muscle rigidity, and severe acidosis, which have been regarded as hallmarks of malignant hyperthermia [5]. It is well-recognized in malignant hyperthermia that tissue hypoxia and muscle breakdown can lead to increased serum creatine kinase levels. However, in this case, the serum creatine kinase level rose modestly to 171 U/L on the third hospital day, which may be considered to be an atypical presentation. This observation might be ascribed to generally lower creatine kinase values in females than males [23] and the association between muscle mass and creatine kinase levels [24]. Notably, this patient exhibited a slender build with a Body Mass Index of 18.6 kg/m². According to the study by Sheila et al. in 2014, approximately 35% of 129 patients diagnosed with malignant hyperthermia exhibited peak creatine kinase values below 1000 [16]. Additionally, the current case did not exhibit severe acidosis or masseter spasm shortly after the administration of succinylcholine, which are commonly observed in malignant hyperthermia [5]. However, these symptoms are not mandatory for the diagnosis of malignant hyperthermia [5]. Regarding acidosis, while our case did not demonstrate severe acidosis, the HCO3- level of 18.2 mmol/L and PCO2 level of 24.2 mmHg in arterial blood gas analysis suggest metabolic acidosis with respiratory compensation [25], a finding consistent with metabolic acidosis observed in malignant hyperthermia [5].

According to Larach et al. 2006, only 9.4% of 181 malignant hyperthermia patients showed disturbance of consciousness [15]. However, this patient experienced a prolonged alteration in consciousness. The extent of consciousness impairment due to malignant hyperthermia considerably varies, with reported cases ranging from a rapid recovery within 90 min [26] to prolonged cognitive disruption exceeding 40 days, ultimately resulting in the sequelae of severe cognitive dysfunction [27]. This case was complicated by dehydration and its associated hyponatremia, which may also have affected the disturbance of consciousness.

Calcium channel blockers we used to treat severe hypertension (over 200 mmHg) might have intensified malignant hyperthermia symptoms by increasing calcium concentrations in skeletal myocytes, along with associated muscle rigidity [1]. A more suitable approach could have employed an antihypertensive agent without calcium channel inhibitory effects.

### Mechanisms of malignant hyperthermia

The underlying mechanisms of malignant hyperthermia involve the excessive release of calcium from the sarcoplasmic reticulum, a crucial calcium store within skeletal muscle cells, into the cytoplasm. This dysregulation is provoked by depolarizing neuromuscular blocking agents, predominantly succinylcholine, and volatile inhalation anesthetics [1]. This disruption results in a marked increase in skeletal muscle metabolic processes, leading to enhanced muscle fiber contraction, increased oxygen consumption, elevated carbon dioxide production, and accelerated depletion of adenosine triphosphate, alongside associated heat production [1]. These physiological changes give rise to the clinical presentations observed, including hyperthermia and muscle rigidity.

Succinylcholine is routinely employed as a muscle relaxant during ECT due to its rapid onset of action and ultra-short duration, making it suitable for the brief anesthesia required in ECT procedures that typically last only a few minutes (Dao et al., 2023) [28]. However, it is important to note that depolarizing muscle relaxants like succinylcholine, along with volatile inhalational anesthetics, are known to trigger malignant hyperthermia [1]. Patients with a known or suspected history of malignant hyperthermia should be administered anesthetics that do not pose a risk of triggering this condition to prevent the onset of malignant hyperthermia. For example, rocuronium, along with its antagonist sugammadex, should be used instead of succinylcholine, as the study by Sumitani et al. (2011) found that the use of rocuronium was not statistically or significantly associated with malignant hyperthermia [29].

### Warning to psychiatrists

Detection and diagnosis of malignant hyperthermia significantly depend on the judgment of psychiatrists. In the case we observed, malignant hyperthermia manifested two hours after ECT, aligning with prior research findings. Visoiu et al. (2014) noted a median time of 76.5 min from the induction of anesthesia to the onset of malignant hyperthermia in cases reported since 1998 [30]. Furthermore, they reported that the upper third quartile duration from the initiation of anesthesia to the onset of malignant hyperthermia was 148.3 min. Given the considerably shorter duration of ECT compared to general surgery, the probability of postoperative malignant hyperthermia after ECT might be higher.

Therefore, malignant hyperthermia after ECT is primarily considered to occur in a psychiatric ward and be detected by psychiatrists, rather than in the operating room by anesthesiologists. It is therefore critically important to closely monitor for indicators of malignant hyperthermia, including abnormal vital signs, such as elevated temperature, tachycardia, high blood pressure, and tachypnea, as well as characteristic physical presentations, such as muscle rigidity, masseter spasm, and cola-colored urine [5], for a few hours after ECT in a psychiatric ward.

## Conclusion

Given the potential lethality of malignant hyperthermia following ECT, it is essential for psychiatrists to rigorously monitor both intraoperative and postoperative vital signs and physical presentations. Identification at the earliest opportunity and timely intervention with dantrolene are crucial in managing malignant hyperthermia.

## Data Availability

The data of this case report are available from the corresponding author (MN) upon request.

## Abbreviations

ECT: Electroconvulsive therapy
MADRS: Montgomery-Åsberg Depression Rating Scale

## References

[1] Rosenberg H, Pollock N, Schiemann A, Bulger T, Stowell K (2015). Malignant hyperthermia: a review. Orphanet J Rare Dis.

[2] Larach MG, Brandom BW, Allen GC, Gronert GA, Lehman EB (2014). Malignant hyperthermia deaths related to inadequate temperature monitoring, 2007–2012: a report from the north American malignant hyperthermia registry of the malignant hyperthermia association of the United States. Anesth Analg.

[3] Lazarus A, Rosenberg H (1991). Malignant hyperthermia during ECT. Am J Psychiatry.

[4] Inada T, Iwamoto K, Takahashi N, Yamamoto N (2013). Mastering the use of MADRS:clinical assessment of depression using the Japanese version of MADRS with SIGMA: revised 3rd edition.

[5] Larach MG, Localio AR, Allen GC, Denborough MA, Ellis FR, Gronert GA, Kaplan RF, Muldoon SM, Nelson TE, Ording H (1994). A clinical grading scale to predict malignant hyperthermia susceptibility. Anesthesiology.

[6] Chemnitz G, Schmidt E, Koller PU, Busch EW. 1979. Kreatinkinase. Uberarbeitete Standardmethode: Referenzwerte und Klinik [Creatine kinase: reference values and clinical aspects of the revised standard method (author’s transl)]. Dtsch Med Wochenschr. 104(7), 257 – 60. German. 10.1055/s-0028-1103881. 10.1055/s-0028-1103881 761520

[7] Wang RZ, Vashistha V, Kaur S, Houchens NW (2016). Serotonin syndrome: preventing, recognizing, and treating it. Cleve Clin J Med.

[8] Tse L, Barr AM, Scarapicchia V, Vila-Rodriguez F (2015). Neuroleptic malignant syndrome: a review from a clinically oriented perspective. Curr Neuropharmacol.

[9] Knadler MP, Lobo E, Chappell J, Bergstrom R (2011). Duloxetine: clinical pharmacokinetics and drug interactions. Clin Pharmacokinet.

[10] Mason PJ, Morris VA, Balcezak TJ (2000). Serotonin syndrome. Presentation of 2 cases and review of the literature. Med (Baltim).

[11] Boyer EW, Shannon M. 2005. The serotonin syndrome. N Engl J Med. 17;352(11), 1112-20. doi: 10.1056/NEJMra041867. Erratum in: N Engl J Med. 2007;356(23), 2437. Erratum in: N Engl J Med. 2009;361(17), 1714. 10.1056/NEJMra041867 15784664

[12] Jones D, Story DA (2005). Serotonin syndrome and the anaesthetist. Anaesth Intensive Care.

[13] Birmes P, Schmitt CD, Lauque L (2003). Serotonin syndrome: a brief review. CMAJ.

[14] White DA, Robins AH (2000). An analysis of 17 catatonic patients diagnosed with neuroleptic malignant syndrome. CNS Spectr.

[15] Larach GM, Gronert AG, Allen CG, Brandom WB, Lehman BE (2006). Clinical presentation, treatment, and complications of malignant hyperthermia in North America from 1987 to 2006. Anesth Analg.

[16] Sheila R, Green ML, Charles H, Duminda W, Christine M, Natalia K (2014). Malignant hyperthermia in Canada: characteristics of Index Anesthetics in 129 malignant Hyperthermia Susceptible Probands. Anesth Analgesia.

[17] Sold M, Tschöp M, Sörensen N (1986). Symptome Der Akuten Einklemmung Nach Narkoseeinleitung Bei Hydrocephalus–in Wahrheit eine maligne hyperthermie [Symptoms of acute cerebral hernia following induction of anesthesia in hydrocephalus–malignant hyperthermia in reality]. Anaesthesist.

[18] Ortiz JF, Wirth M, Eskander N, Cozar JC, Fatade O, Rathod B (2020). The genetic foundations of Serotonin Syndrome, neuroleptic malignant syndrome, and malignant hyperthermia: is there a genetic Association between these disorders?. Cureus.

[19] Tormoehlen LM, Rusyniak DE (2018). Neuroleptic malignant syndrome and serotonin syndrome. Handb Clin Neurol.

[20] Wappler F, Fiege M, Schulte am, Esch J (2001). Pathophysiological role of the serotonin system in malignant hyperthermia. Br J Anaesth.

[21] Litman RS, Flood CD, Kaplan RF, Kim YL, Tobin JR (2008). Postoperative malignant hyperthermia: an analysis of cases from the North American Malignant Hyperthermia Registry. Anesthesiology.

[22] Johnson GC, Santos AB (1983). More on ECT and malignant hyperthermia. Am J Psychiatry.

[23] Neal CR, Ferdinand CK, Ycas J, Miller E (2009). Relationship of ethnic origin, gender, and age to blood creatine kinase levels. Am J Med.

[24] Garcia W (1974). Elevated creatine phosphokinase levels Associated with large muscle Mass another Pitfall in evaluating clinical significance of total serum CPK activity. JAMA.

[25] Castro D, Patil SM, Zubair M, Keenaghan M. 2024. Arterial Blood Gas. In: StatPearls [Internet]. Treasure Island (FL): StatPearls Publishing; 2024 Jan–. (version of 2024 Jan 8). 30725604

[26] Lee YS, Kim WY, Lee SH, Baek SM, Ok SJ, Kim JH, Park YC (2010). A case of malignant hyperthermia during anesthesia induction with sevoflurane -A case report. Korean J Anesthesiol.

[27] Minami S, Ikeda A, Yamada K, Kajihama A, Shimizu H, Nagafuchi H (2023). Pediatric fulminant malignant hyperthermia with severe electroencephalographic abnormality and brain damage: a case report. J Med Case Rep.

[28] Dao QA, Mohapatra S, Kuza C, Moon ST (2023). Traumatic brain injury and RSI is rocuronium or succinylcholine preferred?. Curr Opin Anaesthesiol.

[29] Sumitani M, Uchida K, Yasunaga H, Horiguchi H, Kusakabe Y, Matsuda S, Yamada Y (2011). Prevalence of malignant hyperthermia and relationship with anesthetics in Japan: data from the diagnosis procedure combination database. Anesthesiology.

[30] Visoiu M, Young MC, Wieland K, Brandom BW (2014). Anesthetic drugs and onset of malignant hyperthermia. Anesth Analg.

